# Supplementary material for: NLRX1 Prevents M2 Macrophage Polarization and Excessive Renal Fibrosis in Chronic Obstructive Nephropathy
Source: Cells. 2023 Dec 21;13(1):23. doi: 10.3390/cells13010023 (PMC10778504; doi:10.3390/cells13010023)
Supplement: Supplementary file 1 [file cells-13-00023-s001.zip › cells-2652599-supplementary.pdf]

SUPPLEMENTARY INFORMATION

Supplementary Table S1. Primers for qPCR.

Gene	Full name	Forward primer	Reverse primer
Nlrx1	NOD-like receptor X1	TTGCCATTTGCCCAGGACCTCTT	GGATCAAGAAGGAGATATGCTCATCTGGTAG
Ppia	Peptidylprolyli somerase A	TGCCAGGGTGGTGACTTTAC	GATGCCAGGACCTGTATGCT
Tbp	TATA box binding protein	GGAGAATCATGGACCAGAACA	GATGGGAATTCCAGGAGTCA
Tgfβ	Transforming growth factor beta	GCAACATGTGGAACTCTACCAGAA	GACGTCAAAAGACAGCCACTCA
Col1A1	Collagen-1	ACCTAAGGGTACCGCTGGA	TCCAGCTTCTCCATCTTTGC
Fn1	Fibronectin-1	CCAGAGGAGGCACAAGGTTC	GGGAAACCGTGTAAGGGTCA
Nos2	Nitric oxide synthase	CCAAGCCCTCACCTACTTCC	CTCTGAGGGCTGACACAAGG
Arg1	Arginase 1	CTCCAAGCCAAAGTCCTTAGAG	AGGAGCTGTCATTAGGGACATC
Mgl1	Macrophage galactose type lectin-1	TGAGAAAGGCTTTAAGAACTGGG	GACCACCTGTAGTGATGTGGG
Mrc1	Mannose receptor, C type 1	CTAACTGGGGTGCTGACGAG	GGCAGTTGAGGAGGTTCAGT
Cox4i1	Cytochrome c Oxidase Subunit 4I1	TGGGAGTGTTGTGAAGAGTGA	GCAGTGAAGCCGATGAAGAAC
Ndufa2	NADH dehydrogenase 1	GCACACATTTCCCCACACTG	CCCAACCTGCCCATTCTGAT
Ndufa3	NADH dehydrogenase 3	TACCACAAACGCAGCAAACC	AAGGGACGCCATTAGAAACG
Cyt c	Cytochrome c	TCCATCAGGGTATCCTCTCC	GGAGGCAAGCATAAGACTGG
Cpt1b	Carnitine palmitoyltransferase 1b	CTCCTTTCCTGGCTGAGGTA	GCACCCAGATGATTGGGATA
Acadm	Acyl-CoA dehydrogenase, medium chain	GAAGCCACGAAGTATGCCCT	CCTTCATCGCCATTTCTGCG

Acadl	Acyl-CoA dehydrogenase, long chain	GTAGCTTATGAATGTGTGCAACTC	GTCTTGCGATCAGCTCTTTCATTA
Tfam	Mitochondrial transcription factor A	TCGCATCCCCTCGTCTATCA	CCACAGGGCTGCAATTTTCC
Kim1	Kidney injury molecule 1	TGGTTGCCTTCCGTGTCTCT	TCAGCTCGGGAATGCACAA
Ngal	Neutrophil gelatinase-associated lipocalin	GCCTCAAGGACGACAACATC	CTGAACCATTGGGTCTCTGC
16srRNA	16S ribosomal RNA	CCGCAAGGGAAAGATGAAAGAC	TCGTTTGGTTTCGGGGTTTC
Hk2	Hexokinase 2	GGGAACACAAAAGACCTCTTCTGG	GCCAGCCTCTCCTGATTTTAGTGT
F480 (Emr1)	EGF-like module-containing mucin-like hormone receptor-like 1	CTTTGGCTATGGGCTTCCAGTC	GCAAGGAGGGACAGAGTTTATCGTG
